# Identification of Conserved Regions and Residues within Hedgehog Acyltransferase Critical for Palmitoylation of Sonic Hedgehog

**DOI:** 10.1371/journal.pone.0011195

**Published:** 2010-06-23

**Authors:** John A. Buglino, Marilyn D. Resh

**Affiliations:** 1 Cell Biology Program, Memorial Sloan-Kettering Cancer Center, New York, New York, United States of America; 2 Graduate Program in Biochemistry, Cell and Molecular Biology, Weill Graduate School of Medical Sciences, Cornell University, New York, New York, United States of America; Massachusetts Institute of Technology, United States of America

## Abstract

**Background:**

Sonic hedgehog (Shh) is a palmitoylated protein that plays key roles in mammalian development and human cancers. Palmitoylation of Shh is required for effective long and short range Shh-mediated signaling. Attachment of palmitate to Shh is catalyzed by Hedgehog acyltransferase (Hhat), a member of the membrane bound O-acyl transferase (MBOAT) family of multipass membrane proteins. The extremely hydrophobic composition of MBOAT proteins has limited their biochemical characterization. Except for mutagenesis of two conserved residues, there has been no structure-function analysis of Hhat, and the regions of the protein required for Shh palmitoylation are unknown.

**Methodology/Principal Findings:**

Here we undertake a systematic approach to identify residues within Hhat that are required for protein stability and/or enzymatic activity. We also identify a second, novel MBOAT homology region (residues 196–234) that is required for Hhat activity. In total, ten deletion mutants and eleven point mutants were generated and analyzed. Truncations at the N- and C-termini of Hhat yielded inactive proteins with reduced stability. Four Hhat mutants with deletions within predicted loop regions and five point mutants retained stability but lost palmitoylation activity. We purified two point mutants, W378A and H379A, with defective Hhat activity. Kinetic analyses revealed alterations in apparent K_m_ and V_max_ for Shh and/or palmitoyl CoA, changes that likely explain the catalytic defects observed for these mutants.

**Conclusions/Significance:**

This study has pinpointed specific regions and multiple residues that regulate Hhat stability and catalysis. Our findings should be applicable to other MBOAT proteins that mediate lipid modification of Wnt proteins and ghrelin, and should serve as a model for understanding how secreted morphogens are modified by palmitoyl acyltransferases.

## Introduction

Sonic Hedgehog (Shh) is a secreted morphogen that signals in a concentration dependent fashion [1]. Shh signaling is essential for the proper growth, differentiation and patterning of a variety of tissue types during embryogenesis, including the brain, central nervous system and proximal and distal limb elements[1]–[4]. In addition to its role in development, aberrant Shh signaling has been implicated in the formation and maintenance of multiple human cancers, including medulloblastoma, melanoma, liver, pancreatic, and urogenital tumors [5], [6].

All members of the Hedgehog family undergo a unique series of post-translational processing reactions [7]. Shh is initially synthesized as a 45-kDa precursor protein containing an N-terminal signal sequence which promotes entry into the secretory pathway. Upon cleavage of the signal sequence, the C-terminal Shh autoprocessing domain catalyzes an autocleavage reaction, producing a C-terminal 25-kDa fragment and a 19-kDa N-terminal signaling molecule (ShhN) [8]. Two lipid modifications of ShhN then occur. The newly generated C-terminus of ShhN is modified with cholesterol during the autocleavage reaction [9]. Palmitate is attached via amide linkage to the N-terminal cysteine in a reaction catalyzed by Hedgehog acyltransferase (Hhat) [10]. Hhat mediated Shh palmitoylation can occur independently of autocleavage or cholesterol modification [11].

Palmitoylation of Shh is essential for proper signaling. Mutation of the N-terminal Cys to Ser diminishes Shh patterning activity in the mouse limb and neural tube, and essentially eliminates Hh signaling activity in *Drosophila* [4], [12]–[15]. When tested in an *in vitro* differentiation assay, fatty acylated forms of Shh are significantly more active than non-acylated Shh [10], [15]. The hydrophobic character of palmitate appears to be critical for Shh signaling as chemical modification of the N-terminus with other hydrophobic groups or amino acids can in part rescue signaling by non-acylated forms of Shh [16]. Attachment of cholesterol to the C-terminus of Shh is also important for Shh function, particularly for long range signaling [14], [17]–[20]. Dual lipid modification of Shh has been shown to enhance interaction with lipoprotein particles and formation of soluble multimeric forms of Shh, both of which have been implicated in formation of the Shh signaling gradient and long-range transport throughout tissues [21]–[23].

In a recent study, we reported the purification of Hhat to apparent homogeneity and demonstrated that Hhat is sufficient for palmitoylation of Shh [11]. Hhat is a member of the MBOAT (membrane-bound *O-*acyltransferase) family of multipass transmembrane proteins [24], [25]. MBOAT family members are characterized by the presence of a region of highly conserved residues (MBOAT Homology Domain) within which an invariant histidine residue has been implicated in catalysis [26]–[30]. The majority of MBOAT family members transfer fatty acids and other lipids onto hydroxyl groups of membrane-bound lipids [24], [25], [28], [31], [32]. Examples include enzymes that catalyze phospholipid acyl chain remodeling, formation of cholesterol esters and the formation of cellular stores of triglycerides [25], [27]–[29], [31]–[35]. The most studied MBOAT proteins are the ACATs (Acyl-CoA:Cholesterol Acyltransferases) ACAT1 and ACAT2 that catalyze formation of cholesterol esters. The invariant His is required for the activity of both enzymes. In addition, several other residues/regions have been implicated in protein stability, substrate binding and/or catalytic activity [27], [29], [35], [36]. Recent studies have also identified conserved motifs within MBOAT family members that act as lysophospholipid acyltransferases (LPATs) [33]. However it is not clear which, if any, of these residues/regions would be important in the context of fatty acid transfer to a protein substrate.

Besides Hhat and its *Drosophila* homologue Rasp, only two other MBOAT proteins, Porcupine (Porc) and GOAT (ghrelin O-acyl transferase), transfer fatty acids to proteins. Porc is a putative palmitoylacyltransferase (PAT) implicated in acylation of Wnt/Wg proteins, another family of secreted morphogens. GOAT is the transferase mediating attachment of octanoate to the appetite-stimulating hormone proghrelin [30], [37]–[39]. Apart from highly conserved histidine and aspartate/asparagine residues, the importance of other residues or regions within Hhat, Porc and/or GOAT for catalysis has not been investigated.

In this study, we generated truncations, deletions and point mutations within Hhat in order to identify specific regions and residues required for protein stability and enzymatic activity. We also identified a second region of homology within the MBOAT family members that acylate protein substrates. Mutagenesis of residues within this region compromised Hhat PAT activity *in vitro*. Finally, we purified two Hhat mutants that had expression and stability levels similar to wild-type Hhat, but exhibited decreased PAT activity. These mutants displayed altered kinetic characteristics that may explain their defects in catalysis.

## Materials and Methods

### Reagents and Antibodies

Coenzyme A, CoA synthetase, octylglucoside, anti-Flag and anti-HA antibodies, Flag M2 agarose and 3xFlag peptide were purchased from Sigma (St. Louis, MO). Anti-Shh antibodies were purchased from Santa Cruz Biotechnology. Anti-GFP antibodies were purchased from Roche. [^125^I] NaI was obtained from Perkin Elmer.

### Mammalian expression plasmids, cell culture and transfection

Plasmids encoding HA-tagged and HAFlag6xHis- tagged Hhat, and 1–44 Shh:GFP were generated as previously described [11]; WT Hhat corresponds to GenBank Accession #CAI22284. PCR fragments encoding Hhat HA Δ1–28, Δ1–89, Δ460–493, and Δ429–493 were ligated into the BamHI/EcoRI sites of pcDNA3.1. Hhat HAFlag6xHis Δ153–158, Δ187–192, Δ228–234, Δ313–320, Δ368–380, Δ417–426, S182A, Y207A, G217A, S221A, F338A, D339A, L346A, Y351A, F372A, W378A, H379A constructs were generated by site directed mutagenesis using the Quikchange mutagenesis kit (Stratagene). A plasmid encoding full length human Shh was a generous gift from Dr. Jessica Treisman (New York Univ, NY). All constructs and mutations were confirmed by DNA sequencing. COS-1 cells and 293FT cells (Invitrogen) were grown and maintained as described [11]. Transfections were carried out using Lipofectamine (Invitrogen).

### Synthesis of ^125^ I-iodo-palmitate analogues

Radioiodination of iodo-palmitate with [^125^I] NaI and synthesis of ^125^ I-iodo-palmitoyl CoA using CoA synthetase were performed as previously reported [40], [41]. The final concentration of purified ^125^I-iodo-palmitoyl CoA was determined from the absorbance at 260 nm using the extinction coefficient for palmitoyl-CoA.

### *In Vivo* palmitate labeling

COS-1 cells expressing Shh and either WT or the indicated mutant Hhat construct were starved for 1 hr in DMEM containing 2% dialysed fetal calf serum, followed by incubation with 10–20 µCi/ml [^125^I] iodo-palmiate [41] for 4 hrs at 37°C. Cell lysates were processed and subjected to immunoprecipitation, electrophoresis on 12.5% SDS-PAGE gels, and phosphorimaging as described [11]. Phosphorimaging screens were analyzed on a FLA-7000 phosphorimager (Fuji). Protein levels were determined by SDS-PAGE and Western blot analysis. Labelings were performed in duplicate and repeated three times.

### Expression and purification of recombinant Shh and HhatHAFlagHis

Shh24-197 was purified from recombinant *E. coli* as previously described [11]. For Hhat purification, 10×100 mm plates of 293FT cells were transfected with WT, W378A, or H379A HhatHAFlagHis cDNA or pcDNA3.1 empty vector. 48 hrs post transfection, membrane fractions were generated, solublized and subjected to purification by Flag affinity chromatography as described previously [11]. Samples of the final purified fractions were subjected to SDS-PAGE and Western blotting. Protein concentrations were determined using the DC Protein Assay (Bio Rad).

### *In vitro* palmitoylation assay

10 µl of total cell lysate (20 µg), or a P100 membrane fraction (10 µg) generated from cells transfected with the indicated Hhat constructs [11], was combined with 10 µl of recombinant Shh (0.2 mg/ml in 20 mM MES, pH 6.5, 1 mM EDTA, 1 mM DTT), followed by the addition of 30 µl of reaction buffer (167 mM MES, pH 6.5, 1.7 mM DTT, 0.083% Triton X-100, 167 µM ^125^I-iodo-palmitate CoA). After 1 hr at room temperature (unless otherwise indicated), the reaction was stopped by addition of 50 µl of 2× sample buffer with 40 mM DTT. Samples were electrophoresed on 12.5% SDS-PAGE gels, stained with Coomassie Blue, dried and exposed to a phosphorimager screen for 12–18 hrs. Each Shh containing gel band was then excised and ^125^I-iodo-palmitate incorporation was measured by counting in a Perkin-Elmer Gamma counter. Non-enzymatic incorporation of ^125^I-iodo-palmitate into Shh was corrected for by subtraction of counts from matched pcDNA 3.1 mock controls.

### Determination of apparent K_m_ and V_max_ values

10 µl of purified WT, W378A, and H379A HhatHAFlag6xHis (∼20 ng) in elution buffer (20 mM HEPES [pH 7.3], 100 mM NaCl, 1% octylglucoside, 1% glycerol) were reacted with saturating concentrations of either recombinant Shh (40 µM) or ^125^I-iodo-palmitate CoA (100 µM) in the presence of the indicated concentrations of the other substrate for 30 min at room temperature. The reaction mix was separated and quantified as described above. K_m_ and V_max_ values were determined by non-linear regression using the enzyme kinetic module in Graph Pad Prism.

### Protein Stability Assay

COS-1 cells transfected with the indicated Hhat constructs were split into 60 mm dishes and incubated at 37°C for 24 hrs. The cells were placed in DMEM media supplemented with 10% FBS, 100 µg/ml cycloheximide, and 40 µg/ml chloramphenicol and incubated for the indicated times. Cells were washed 2× in 2 ml of STE and scraped in 500 µl of 2xSB containing 40 mM DTT. Samples were electrophoresed on 12.5% SDS-PAGE gels, transferred onto PVDF membranes and probed with anti-HA antibody to determine protein levels.

### Bioinformatics

The Kyte-Doolittle plot of Hhat hydrophobicity was generated using the website: http://www.vivo.colostate.edu/molkit/hydropathy/index.html. Multiple sequence alignment was carried out using the TCoffee alignment program. The graphics were modified from images generated by the Swiss Institute of Bioinformatics website: http://tcoffee.vital-it.ch/cgi-bin/Tcoffee/tcoffee_cgi/index.cgi?stage1=1&daction=TCOFFEE::Regular. Hhat membrane topology prediction was performed via the TMHMM Server v. 2.0: http://www.cbs.dtu.dk/services/TMHMM-2.0/.

## Results

### N- and C-terminal truncation mutants of Hhat lack palmitoylation activity and exhibit reduced protein stability

**Figure 1:**
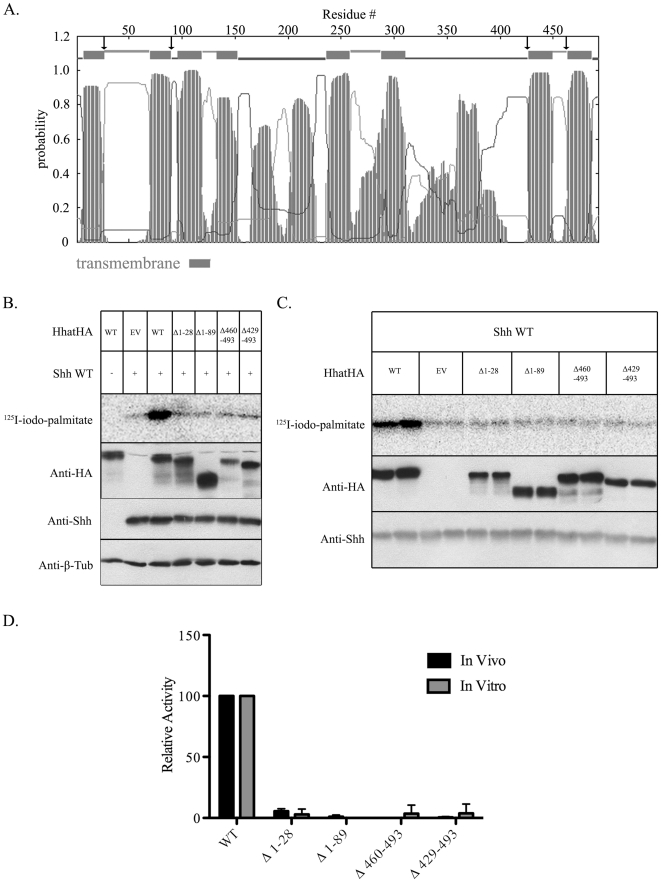
N and C Terminal truncation mutants of Hhat lack PAT activity. **A.** Transmembrane topology model of Hhat generated using the TMHMM v. 2.0 Server. Gray bars indicate predicted transmembrane helices. Arrows denote approximate truncation points. **B.** COS-1 cells were transfected with the indicated constructs and labeled with ^125^I-iodo-palmitate for 4h. Cell lysates were analyzed directly by Western blotting or after immunoprecipitation of Shh. Upper panel: ^125^I-iodo-palmitate incorporation into immunoprecipitated Shh as detected by phosphorimaging. Lower panels: Western blots of the same extracts probed with anti-HA, anti-Shh, and anti-β-tubulin (β-Tub) antibodies. EV, empty vector. **C.** An *in vitro* palmitoylation assay was performed with P100 membranes generated from 293FT cells transfected with WT and mutant Hhat constructs. Upper panel: ^125^I-iodo-palmitate incorporation into Shh detected by phosphorimaging. Lower panels: Western blots of the same samples probed with anti-HA and anti-Shh antibodies. **D.** Quantification of the experiments in panels B and C. Levels of ^125^I-iodo-palmitate incorporation were corrected for Hhat protein expression and normalized to WT Hhat levels (100%). Each bar represents the average of three experiments and is expressed as the percent of WT activity (set to 100%).

In an attempt to isolate a minimal domain required for Hhat palmitoyl acyltransferase (PAT) activity, we engineered mutant constructs of Hhat truncated at either the N or C terminus. Truncation points were chosen based on transmembrane topology modeling and were predicted to delete one or two transmembrane segments from either end of the Hhat polypeptide (Fig. 1A). COS-1 cells were co-transfected with cDNAs encoding Shh and either wild type (WT) or mutant Hhat, and labeled with ^125^I-Iodopalmitate, a radioiodinated palmitate analog [40]. Shh was immunoprecipitated from cell lysates, and the amount of radiolabeled palmitate incorporated into Shh was determined by phosphorimaging analysis after SDS-PAGE. The truncation mutants were expressed at levels similar to WT Hhat, but none of the mutants promoted Shh palmitoylation above levels achieved with mock-transfected (empty vector) controls (Fig. 1B, D).

We next prepared membrane fractions from cells expressing either WT Hhat or the truncation mutants, and analyzed PAT activity in an *in vitro* Shh palmitoylation assay. The assay consists of membranes from Hhat transfected cells, purified recombinant Shh protein, and ^125^I-Iodopalmitoyl CoA, and monitors incorporation of ^125^I-Iodopalmitate into Shh (11). None of the truncation mutants were able to support Shh palmitoylation above control levels (Fig. 1C, D). Analysis by indirect immunofluorescence and confocal imaging revealed that the subcellular localization pattern of each of the four truncation mutants was indistinguishable from WT Hhat (data not shown), suggesting that the defect in Hhat PAT activity observed was not due to gross mislocalization of the mutant proteins.

**Figure 2:**
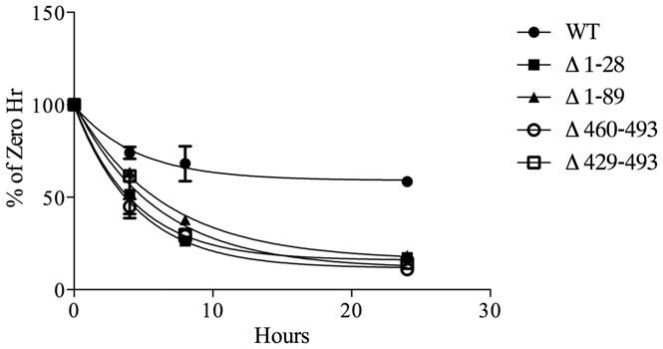
Stability of Hhat truncation mutants. COS-1 cells transfected with the indicated Hhat constructs were incubated in DMEM supplemented with 10% FBS, 100 µg/ml cycloheximide, and 40 µg/ml chloramphenicol. At each indicated time point, cells were lysed and subjected to SDS-PAGE and Western blotting with anti-HA antibodies. The amount of HA signal at each time point was determined using ImageJ software. Data are expressed as percent of 0 h controls, which were set to 100%. Experiments were carried out in duplicate and repeated three times. Values for the percentage of Hhat protein remaining at 24 h were: WT, 58%; Δ1–28, 17%, Δ1–89 19%, Δ460–493 11%, Δ429–493 14%. Estimated half-lives for the mutants were 3–7 h, compared to >20 h for WT Hhat.

Loss of enzyme activity could be the result of altered protein folding and/or stability. In order to address this possibility, Hhat transfected COS-1 cells were treated with cycloheximide, to block new protein synthesis, and levels of WT and Hhat truncation mutant proteins were monitored as a function of time. WT Hhat appeared to be quite stable, with approximately 60% of the initial level remaining 24 hrs after cycloheximide addition (Fig. 2). By contrast, in the absence of ongoing protein synthesis, all four truncation mutants exhibited reduced stability, with less than 20% of initial levels of mutant Hhat protein remaining after 24 hrs (Fig. 2). These findings suggest that the N- and C-terminal truncation mutants are misfolded, and that this defect might account for the deficiency in PAT activity, although other explanations are also possible.

### Mutational analysis of predicted loop regions

**Figure 3:**
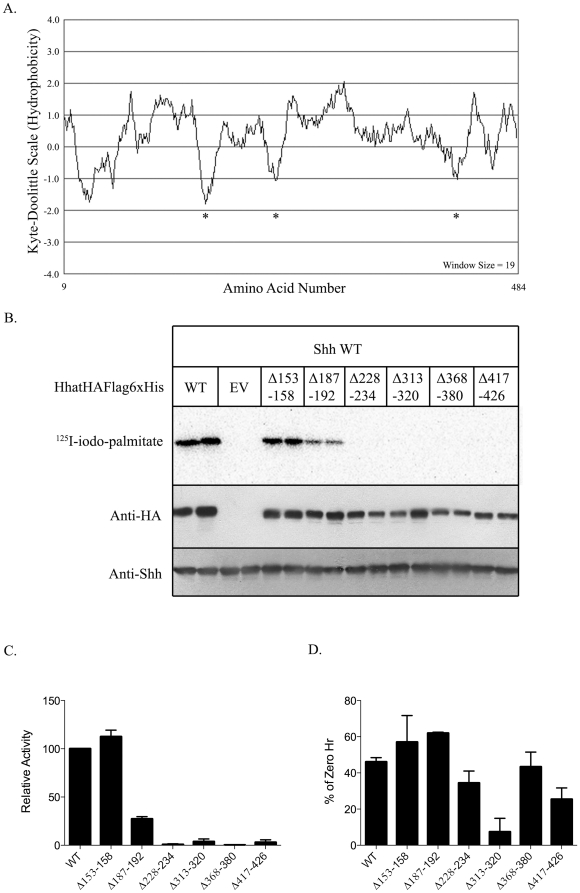
PAT activity and stability measurements of Hhat constructs containing deletions within predicted loop regions. **A.** Kyte-Doolittle hydropathy plot of Hhat with the window size set at 19. Asterisks indicate regions of high hydrophilicity that were targeted for mutagenesis. **B.** Representative *in vitro* palmitoylation assay. P100 membranes isolated from 293FT cells expressing the indicated Hhat constructs were reacted with Shh and ^125^I-iodo-palmitate CoA as described in Materials and Methods. EV, empty vector. **C.** Quantification of the palmitoylation assays performed three times. Levels of ^125^I-iodo-palmitate incorporation were corrected for Hhat protein expression and normalized to WT Hhat (100%). Data are expressed as a percent of WT activity. **D.** Relative stability of Hhat deletion mutants after 24 h incubation with cycloheximide and chloramphenicol, as described in Figure 2. Data are expressed as a percentage of 0 h controls. Experiments were performed in duplicate and repeated three times.

Transmembrane topology modeling of Hhat predicts two large loop regions between residues 153–235 and 311–426 (Fig. 1A). In order to address the functional requirement for amino acids within one or both of these potential loops, we generated constructs with residues deleted within one or the other regions. Deletions were specifically targeted to residues/regions of Hhat predicted to be relatively hydrophilic as judged by the Kyte-Doolittle hydropathy scale (Fig. 3A). We then assayed these deletion mutants for their ability to catalyze Shh palmitoylation in *in vitro* palmitoylation assays.

The majority of the Hhat deletion mutants were compromised for PAT activity towards Shh. However, two deletions within the predicted loop between residues 153 and 235 retained either full (Δ153–158), or partial (Δ187–192) PAT activity, suggesting that these residues are not strictly required for activity (Fig. 3B,C). We next compared the stability of the deletion mutants to that of WT Hhat. As expected, the Δ153–158 mutant, which had PAT activity equivalent to WT, was also as stable as WT Hhat (Fig. 3D, Table 1). One mutant, Δ313–320, was considerably less stable than WT with approximately 90% of the protein degraded after 24 hrs (Fig. 3D, Table 1). However, the other deletion mutants that had partial (Δ187–192) or complete loss of activity (Δ228–234, Δ368–380, Δ417–426) displayed stability similar to or within 45–75% of WT Hhat (Fig. 3D). Thus, deletion of these regions does not dramatically affect protein stability and instead may alter substrate binding and/or catalysis.

**Table 1:** PAT activity and stability measurements of Hhat deletion mutants.

Hhat	PAT Activity^a^		Stability^b^	
	Relative Activity	SEM	% remaining at 24 hr	SEM
WT (HA)	100	0	58.5	0.2
Δ 1–28	2.9	2.2	17.3	1.3
Δ 1–89	0	0	18.6	0.2
Δ 460–493	3.5	3.5	10.9	0.6
Δ 429–493	3.8	3.8	14.3	0.9
WT (HAFlag6XHis)	100	0	46.1	2.3
Δ 153–158	112.5	6.9	57	14.6
Δ 187–192	27.3	2.4	62	0.5
Δ 228–234	0.8	0.8	34.5	6.6
Δ 313–320	3.8	2.8	7.5	7.5
Δ 368–380	0.3	0.3	43.4	8.2
Δ 417–426	3	2.7	25.5	6.2
S182A	104.5	0.6	ND	
Y207A	1.8	0.5	2.6	2.6
G217A	19.8	2.8	35.5	9.7
S221A	87	2	ND	
F338A	32.5	4.5	49.6	6.7
D339A	9.8	6.2	44.5	7
L346A	111.5	16.5	ND	
Y351A	122	9	ND	
F372A	105.7	4.3	ND	
W378A	30	4.4	53.3	3.3
H379A	49.3	6.2	49.6	2.2

a Assays were performed using P100 membranes generated from transfected 293FT cells.

b Relative stability of Hhat deletion mutants after 24 hr incubation with cycloheximide and chloramphenicol, as described in Figure 2. Data is expressed as a percentage of zero hr controls.

All experiments were performed in duplicate and repeated three times.

### Identification of critical conserved residues by alanine scanning mutagenesis

**Figure 4:**
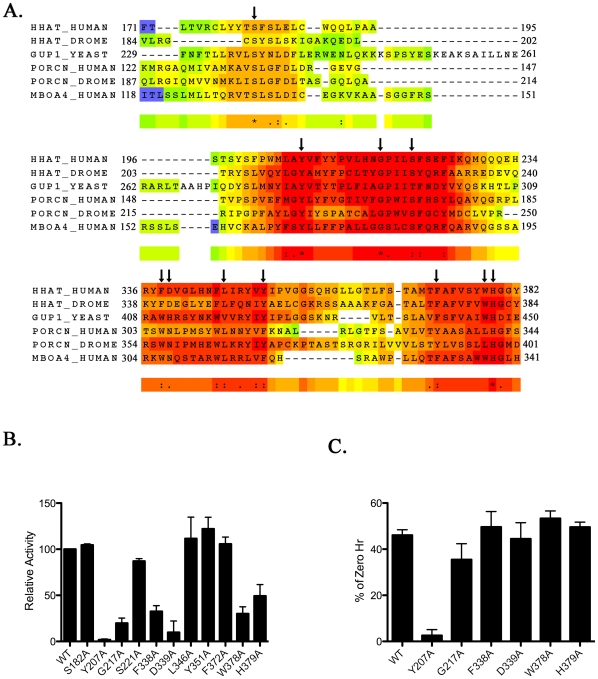
Identification of MBOAT Homology Regions. **A.** Multiple sequence alignment of MBOAT family members that acylate protein substrates generated using the TCoffee server. The sequence from GUP-1/Hhat-like protein was included based on its high homology to Hhat and the functional characterization of GUP-1 from the yeast *S. cerevisiae* [26]. Arrows indicate residues mutated to alanine. **B.** Quantification of *in vitro* palmitoylation assays performed three times. Levels of ^125^I-iodo-palmitate incorporation were corrected for Hhat protein expression and normalized to WT Hhat (100%). Data is expressed as a percent of WT activity. **C.** Relative stability of Hhat point mutants after 24 h incubation with cycloheximide and chloramphenicol. Data are expressed as percent of 0 h controls. Experiments were carried out in duplicate and repeated three times.

One of the hallmarks of the MBOAT family of acyltransferses is the presence of two highly conserved residues, Asp/Asn (position 339 in Hhat) and His (position 379 in Hhat) within the MBOAT homology region, that have been shown to be required for activity in other MBOAT family members [24], [29], [30]. In addition to these residues, previous studies have identified several highly conserved hydrophobic residues whose role in Hhat PAT activity has not been explored [42]–[44]. In an attempt to identify novel residues required for recognition and palmitoylation of protein substrates, we performed a global sequence alignment of MBOAT family members with known protein substrates – Hhat, Porc and GOAT. We included GUP1 (Hhat-like protein), an MBOAT protein involved in GPI-anchor remodeling [26] that exhibits high homology to Hhat (Fig. 4A). In addition to the MBOAT homology region previously reported, we identified a second area of high conservation located between residues 196 and 234 of Hhat (Fig. 4A). To address the importance of this region, as well as the previously reported hydrophobic residues, we mutated eleven of these residues to alanine (arrows in Fig. 4A) and assayed the effect on Hhat PAT activity.

The point mutants can be separated into three groups based on their effects on Hhat activity. Five of the mutations did not substantially affect Hhat activity (S182A, S221A, L346A, Y351A, F372A) indicating that the targeted residues are not required for PAT activity (Fig. 4B). The second group of mutants retained partial (20–50% of WT) activity (G217A, F338A, W378A, H379A). The finding that the H379A mutation causes an approximately 50% reduction in Hhat PAT activity agrees with our previous report [11]. Two mutants (Y207A, D339A) were severely affected (<10% of WT activity) (Fig. 4B). When we compared the relative stability of the point mutants to WT Hhat, only the Y207A mutation affected Hhat stability, with a 95% reduction in protein level after 24 hrs (Fig. 4C). Taken together, these analyses identify five residues that likely contribute to the PAT activity of Hhat.

### Enzymatic characterization of Hhat mutants reveals defects in catalysis

**Figure 5:**
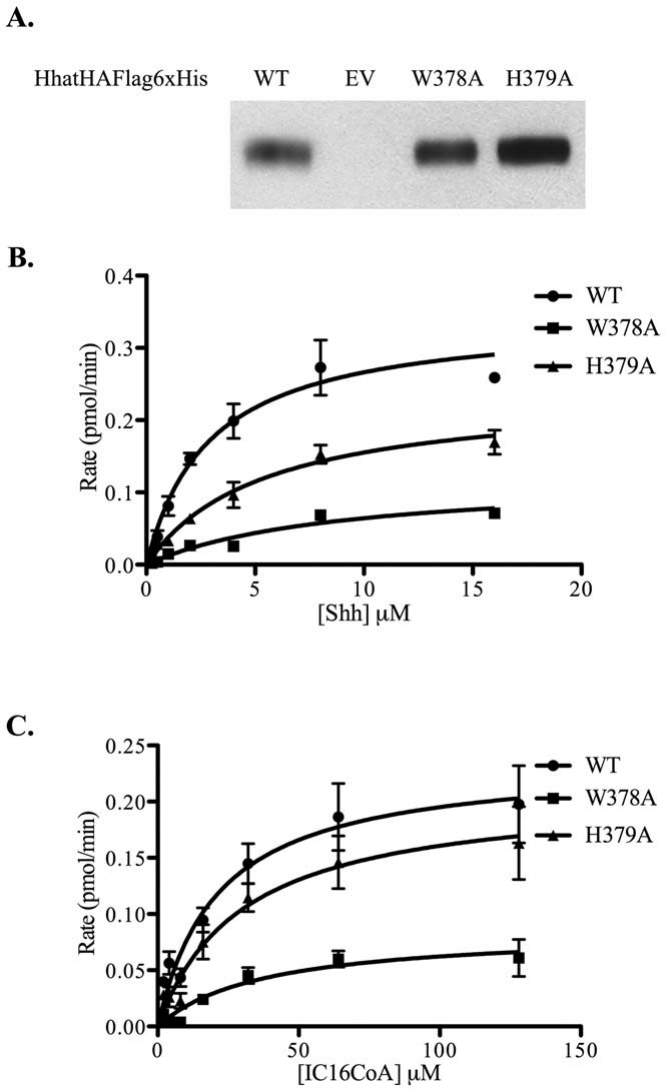
Kinetic analyses of purified Hhat mutants. **A.** The indicated Hhat constructs were expressed in and purified from 293FT cells as described in Materials and Methods. Aliquots of each purified protein were analyzed by SDS-PAGE and Western blotting using anti-Flag antibody. EV, empty vector. **B.** Each purified HhatHAFlagHis construct was incubated with Shh at the indicated concentration in the presence of 100 µM ^125^I-iodo-palmitoyl CoA for 1 h at room temperature. **C.** Each purified HhatHAFlagHis construct was incubated with ^125^I-iodo-palmitoyl CoA at the indicated concentration in the presence of 40 µM Shh. Shh protein bands were excised from dried gels and the amount of ^125^I-iodo-palmitate incorporation was determined by gamma counting. Graphs represent the average of three experiments corrected for non-specific incorporation of ^125^I-iodo-palmitate as described previously [11].

We next performed experiments to identify a mechanism to explain how Hhat mutations altered PAT activity. We chose Hhat constructs with mutations that affected PAT activity without compromising stability. Our working hypothesis is that some mutations will affect the ability of Hhat to bind Shh and/or palmitoyl CoA. First, both co-immunoprecipitation and pulldown assays were performed to monitor Hhat interactions with Shh and palmitoyl CoA, but we were unable to detect stable or specific interactions with either substrate. We therefore performed direct kinetic analyses to compare the apparent K_m_ and V_max_ of candidate mutants to WT Hhat. Candidates were selected that had expression and stability levels similar to WT, and retained more than 10% of WT activity. Five mutants met these criteria: Δ187–192, G217A, F338A, W378A, and H379A. The yield of three of these mutants, Δ187–192, G217A, F338A, was several fold lower than WT Hhat. However, we succeeded in purifying W378A and H379A to levels similar to WT with sufficient yield to carry out kinetic analyses (Fig. 5A).

Mutation of W378 caused a 3-fold reduction in the apparent V_max_ and a 2–3 fold increase in the apparent K_m_ for both Shh and Iodopalmitoyl CoA substrates (Fig. 5B,C and Table 2). These alterations could explain the defect in PAT activity observed for this mutant. By contrast, the H379A mutant exhibited apparent K_m_ and V_max_ values for Iodopalmitoyl CoA that were within 1.1–1.4-fold those of WT Hhat. This mutant appears to bind palmitoyl CoA with similar affinity to WT Hhat when Shh levels are high (40 µM). However, Hhat H379A exhibited a clear defect in catalysis when Shh levels were limiting (Fig. 5B, C and Table 2), suggesting that H379 may play a role in binding of Shh to the enzyme.

**Table 2:** Kinetic analyses of purified Hhat mutants.

Hhat	Shh^a^		Palmitoyl CoA^b^	
	K_m_ (µM)	V_max_ (pmol/min)	K_m_ (µM)	V_max_ (pmol/min)
WT	2.9	0.34	21	0.24
W378A	8.8	0.12	37	0.09
H379A	5.4	0.24	30	0.21

K_m_ and V_max_ values represent best fit values generated by nonlinear regression of the data in Figure 5A, B using Graph pad Prism software.

a Shh titration was performed at 100 µM [^125^I]Iodopalmitoyl CoA.

b Palmitoyl CoA titration was performed at 40 µM Shh.

## Discussion

The presence of multiple transmembrane domains has hampered biochemical studies of MBOAT acyltransferases in general, and Hhat in particular. With the exception of two residues that have been shown to be required for enzymatic activity, there has been no structure-function analysis of Hhat. In this study, we identify specific regions and multiple residues within Hhat that regulate protein stability and/or catalysis. Of note, alignment of the sequences of MBOAT proteins that acylate protein substrates revealed the presence of an additional region of high sequence conservation (Fig. 4) that had not been previously identified. Here we report the results of our analyses of 10 deletion mutants and 11 point mutants within Hhat.

Many of the mutants exhibited increased rates of protein degradation compared to WT Hhat, and nearly all of the mutants in this class had defects in Shh palmitoylation activity. This was particularly evident when truncations were made at the N- or C-terminus of Hhat. However, steady state levels of these mutants, as detected by anti-HA Western blotting in the absence of cycloheximide, appeared to be similar to that of WT Hhat. We quantified the rate of synthesis of Hhat using Tran-^35^S-labeling, and found no change in the rate of synthesis of the mutants compared to WT Hhat (data not shown). One possible explanation to reconcile the observed differences in stability is to postulate that ongoing protein synthesis is required to maintain mutant Hhat protein levels. If the Hhat truncation mutants are misfolded, they could be present at equivalent levels to WT Hhat but would likely be more susceptible to degradation, especially when protein levels are not replenished (ie in the presence of cycloheximide). In this case, misfolding of the mutant proteins might account for the decreased Hhat activity. Alternatively, the truncation mutants could be inactive because regions involved in substrate recognition or catalysis were deleted.

Most of the internal deletion and point mutants were as or nearly as stable as WT Hhat but had reduced PAT activity. In Hhat, these include deletions of residues 187–192, 228–234, and 368–380, as well as the point mutants F338A, D339A, W378A, and H379A. The equivalents of residues F338 and D339 are moderately conserved in the MBOAT family (FD in Hhat and GUP1, FN in LPAT5, and WN in the other family members). W378 and H379 are present in all MBOAT family members, except for GUP1 (Leu in place of His) and Porc and ACAT1 and 2 (Leu or Val in place of Trp). Mutations of residues corresponding to H379A or W378A have been reported in both LPAT and ACAT family members. Mutating either residue abolishes activity in all LPAT family members tested [33]. The conserved His is also absolutely required for ACAT activity [27], [29]. However, mutation of the Val residue at the position corresponding to W378 compromises not only enzymatic activity but also protein expression, complicating its analysis [27].

In addition to the canonical MBOAT homology domain, we also identified residues in a second region (residues 196– 234) that are highly conserved in family members that transfer fatty acids onto protein substrates. Of these, the Tyr at position 207 is also conserved in both LPAT and ACAT family members, whereas the Gly at position 217 is conserved among LPAT but not ACAT family members. In ACAT family members the residue at this position is either Ala or Cys. To date there are no other reports of mutation within this region of another MBOAT family member. It will be interesting to see if residues within this region are important specifically for transfer of fatty acids onto proteins or if they are more broadly required for activity within the MBOAT family.

A prior study reported that an Hhat construct with both D339 and H379 mutated to Ala was not able to rescue the phenotype of an Hhat-defective mutant [42]. We have analyzed the effects of each of these mutations separately. D339A Hhat was essentially inactive (<7% of WT activity). H379 has been proposed to be part of the active site of MBOAT proteins. Mutation of this conserved Histidine residue completely abrogates activity for all tested members of the MBOAT family leading to the stipulation that it is directly involved in catalysis. However, the H379A mutant retains 50% of the activity of WT Hhat, suggesting that this residue is not absolutely required for catalysis. Kinetic analyses performed on purified H379A Hhat revealed that this mutant binds palmitoyl CoA with an affinity similar to WT Hhat (Fig. 5). This suggests that H379 may be more important for recognition and binding of Shh. Mutation of the adjacent residue, W378, caused a more severe effect on Hhat activity. W378A Hhat exhibited alterations in apparent K_m_ and V_max_ for both Shh and Iodopalmitoyl CoA substrates. Given the effect on both parameters it is not clear whether the W378A mutant is compromised in catalytic activity, has a severe defect in substrate binding, or a combination of the two. Direct measurements of substrate binding will be required to determine which is the case.

One of the hallmarks of the palmitoylation reaction catalyzed by the other family of PATs, DHHC PATs, is their ability to autoacylate [45]. By contrast, we have not detected acyl-enzyme formation for Hhat and palmitate. Co-immunoprecipitation and pulldown assays aimed at monitoring Hhat interactions with Shh and palmitoylCoA were performed using full length Shh, recombinant ShhN purified from *E.coli*, as well as a biotinylated Shh peptide that we have previously shown acts as a Hhat substrate *in vitro* [11]. We were unable to detect stable or specific interactions of Hhat with any of these substrates. This is not surprising given the hydrophobic nature of the players involved and the fact that enzymes are not expected to bind with high affinity to their substrates as this would tend to hinder enzymatic turnover. Thus, we have not been able to utilize direct binding assays to quantify the interactions between Hhat and its two substrates. Instead, we purified two Hhat mutants, W378A and H379A, to apparent homogeneity, and showed that these mutants exhibited kinetic alterations that may explain their catalytic defects as described above.
